# Bifidobacteria and Their Role as Members of the Human Gut Microbiota

**DOI:** 10.3389/fmicb.2016.00925

**Published:** 2016-06-15

**Authors:** Amy O'Callaghan, Douwe van Sinderen

**Affiliations:** Alimentary Pharmabiotic Centre and School of Microbiology, University College CorkCork, Ireland

**Keywords:** *Bifidobacterium*, carbohydrate metabolism, genetic modification, probiotics, microbe-host interaction

## Abstract

Members of the genus *Bifidobacterium* are among the first microbes to colonize the human gastrointestinal tract and are believed to exert positive health benefits on their host. Due to their purported health-promoting properties, bifidobacteria have been incorporated into many functional foods as active ingredients. Bifidobacteria naturally occur in a range of ecological niches that are either directly or indirectly connected to the animal gastrointestinal tract, such as the human oral cavity, the insect gut and sewage. To be able to survive in these particular ecological niches, bifidobacteria must possess specific adaptations to be competitive. Determination of genome sequences has revealed genetic attributes that may explain bifidobacterial ecological fitness, such as metabolic abilities, evasion of the host adaptive immune system and colonization of the host through specific appendages. However, genetic modification is crucial toward fully elucidating the mechanisms by which bifidobacteria exert their adaptive abilities and beneficial properties. In this review we provide an up to date summary of the general features of bifidobacteria, whilst paying particular attention to the metabolic abilities of this species. We also describe methods that have allowed successful genetic manipulation of bifidobacteria.

## Introduction

The past 20 years has seen a research focus on those members of the gut microbiota that exhibit health-promoting or probiotic effects such as protection of the host against pathogens by competitive exclusion (Bernet et al., 1994; Hooper et al., 1999), modulation of the immune system (O'Hara and Shanahan, 2007), and provision of nutrients through the breakdown of non-digestible dietary carbohydrates (Roberfroid et al., 1995; Leahy et al., 2005). Furthermore, compositional alterations of the gastrointestinal tract (GIT) microbiota have been linked to certain gastrointestinal diseases such as inflammatory bowel disease (Ott et al., 2004) and necrotizing enterocolitis (De La Cochetiere et al., 2004). Particular interest has focused on members of the genus *Bifidobacterium,* some of which have been included as live components in a variety of so-called functional foods (Ventura et al., 2004). Bifidobacteria were first isolated from the feces of breast-fed infants in 1899 by Tissier and since then bifidobacteria have been isolated from a range of different ecological niches such as the oral cavity, sewage and the insect gut, the GIT of various mammals and more recently from water kefir (Klijn et al., 2005; Ventura et al., 2007; Laureys et al., 2016).

Although, it has been well established that bifidobacteria confer positive health benefits to the human host, there is a clear lack of knowledge concerning the molecular mechanisms that explain these probiotic traits of *Bifidobacterium* (Cronin et al., 2011). Deciphering whole genome sequences can shed light on the genetic basis of the probiotic action of bifidobacteria, or indeed the associated molecular adaptations that allow this gut commensal to take up residency in its highly competitive ecological niche (Ventura et al., 2014). Although, a significant sequencing effort of bifidobacterial genomes has generated a very extensive set of genomic data, yet this genomic information has hardly been explored at the functional level due to a lack of tools to make bifidobacteria genetically accessible (Serafini et al., 2012).

## General features of bifidobacterial genomes

Since the publication of the first bifidobacterial genome in 2002, there has been a steady increase in the number of publicly available bifidobacterial genome sequences (Lee et al., 2008). The NCBI data base currently (April 2016) holds 254 publicly available bifidobacterial genome sequences, of which sixty one represent complete genome sequences (Table 1, source; http://www.ncbi.nlm.nih.gov/Taxonomy/Browser/wwwtax.cgi?id=1678NCBI, April 2016). Three or more complete genome sequences are available for certain bifidobacterial species, such as for *B. adolescentis, B. animalis, B. breve, B. bifidum, B. longum,* and *B. angulatum* (Table 1).

**Table 1:** **Summary of all completely sequenced bifidobacterial genomes**.

**Microorganism**	**Genome Size (Mb)**	**Number of genes**	**G+C content (%)**	**tRNA**	**rRNA**	**GenBank**
*B.actinocoloniiforme* DSM 22766	1.83	1502	62.7	47	6	CP011786.1
*B.adolescentis* ATCC 15703	2.09	1721	59.2	54	16	AP009256.1
*B.adolescentis* 22L	2.2	1798	59.3	54	13	CP007443.1
*B.adolescentis* BBMN23	2.17	1812	59.3	55	13	CP010437.1
*B.angulatum* DSM20098	2.02	1615	59.4	53	12	AP012322.1
*B.angulatum* GT102	2.06	1651	59.3	53	3	CP014241.1
*B.animalis* subsp. *lactis* AD011	1.93	1615	60.5	52	7	CP001213.1
*B.animalis* subsp. *lactis* BI-04	1.94	1608	60.5	52	12	CP001515.1
*B.animalis* subsp. *lactis* DSM10140	1.94	1607	60.5	51	12	CP001606.1
*B.animalis* subsp. *lactis* BB-12	1.94	1611	60.5	52	12	CP001853.1
*B.animalis* subsp. *lactis* V9	1.94	1610	60.5	52	12	CP001892.1
*B.animalis* subsp. *lactis* CNCM I-2494	1.94	1611	60.5	52	12	CP002915.1
*B.animalis* subsp. *lactis* BLC1	1.94	1608	60.5	52	12	CP003039.2
*B.animalis* subsp. *animalis* ATCC25527	1.93	1583	60.5	52	11	CP002567.1
*B.animalis* subsp. *lactis* B420	1.94	1610	60.5	52	12	CP003497.1
*B.animalis* subsp. *lactis* Bi-07	1.94	1608	60.5	52	12	CP003498.1
*B.animalis* subsp. *lactis* BI12	1.94	1608	60.5	52	12	CP004053.1
*B.animalis* subsp. *lactis* ATCC27673	1.96	1624	60.6	52	12	CP003941.1
*B.animalis* RH	1.93	1606	60.5	52	8	CP007755.1
*B.animalis* subsp. *lactis* KLDS2.0603	1.95	1610	60.5	52	15	CP007522.1
*B.animalis* A6	1.96	1623	60.5	52	16	CP010433.1
*B.animalis* subsp. *lactis* BF052	1.94	1608	60.5	52	12	CP009045.1
*B.*asteroides PRL2011	2.17	1727	60.1	45	6	CP003325.1
*B.bifidum* PRL2010	2.21	1791	62.7	52	9	CP001840.1
*B.bifidum* S17	2.19	1819	62.8	53	9	CP002220.1
*B.bifidum* BGN4	2.22	1832	62.6	52	9	CP001361.1
*B.bifidum* ATCC29521	2.21	1838	62.7	54	6	AP012323.1
*B.bifidum* BF3	2.21	1813	62.6	52	9	CP010412.1
*B.breve* UCC2003	2.42	2049	58.7	54	6	CP000303.1
*B.breve* ACS-071-V-Sch8b	2.33	1956	58.7	53	9	CP002743.1
*B.breve* 12L	2.24	1883	58.9	52	6	CP006711.1
*B.breve* JCM7017	2.29	1916	58.7	54	6	CP006712.1
*B.breve* JCM7019	2.36	2045	58.6	56	6	CP006713.1
*B.breve* NCFB2258	2.32	1946	58.7	53	6	CP006714.1
*B.breve* 689b	2.33	1970	58.7	53	6	CP006715.1
*B.breve* S27	2.29	1926	58.7	53	9	CP006716.1
*B.breve* DSM20213	2.27	1973	58.9	53	6	AP012324.1
*B.breve* BR3	2.42	2232	59.1	54	9	CP010413.1
*B.catenulatim* DSM16992	2.08	1717	56.2	55	16	AP012325.1
*B.coryneforme* LMG18911	1.76	1423	60.5	46	9	CP007287.1
*B.dentium* Bd1	2.64	2177	58.5	56	13	CP001750.1
*B.dentium* JCM1195	2.64	2177	58.5	56	13	AP012326.1
*B.indicum* LMG11587	1.73	1403	60.5	47	9	CP006018.1
*B.kashiwanohense* PV20-2	2.37	2007	56.1	58	16	CP007456.1
*B.kashiwanohense* JCM15439	2.34	1965	56.3	54	16	AP012327.1
*B.longum* NCC2705	2.26	1797	60.1	57	12	AE014295.3
*B.longum* DJO10A	2.38	1998	60.1	58	12	CP000605.1
*B.longum* subsp. infantis ATCC15697	2.83	2594	59.9	79	12	CP001095.1
*B.longum* subsp. *longum* JDM301	2.48	2062	59.8	55	9	CP002010.1
*B.longum* subsp. *longum* BBMN68	2.27	1873	59.9	54	9	CP002286.1
*B.longum* subsp. *longum* JCM1217	2.39	2001	60.3	73	12	AP010888.1
*B.longum* subsp. infantis 157F	2.4	2044	60.1	59	12	AP010890.1
*B.longum* subsp. *longum* KACC91563	2.39	1979	59.8	56	9	CP002794.1
*B.longum* BXY01	2.48	2065	59.8	55	9	CP008885.1
*B.longum* subsp. *longum* GT15	2.34	1947	60	56	14	CP006741.1
*B.longum* 105-A	2.29	1874	60.1	56	12	AP014658.1
*B.longum* subsp. infantis BT1	2.58	2399	59.4	56	9	CP010411.1
*B.longum* BG7	2.45	2116	60	57	9	CP010453.1
*B.longum* subsp. *longum* NCIMB8809	2.34	1959	60.1	56	9	CP011964.1
*B.longum* subsp. *longum* CCUG30698	2.45	2106	60.2	57	6	CP011965.1
*B.pseudocatenulatum* DSM20438	2.31	1864	56.4	54	19	AP014658.1
*B.pseudolongum* PV8-2	2.03	1704	63.3	53	12	CP007457.1
*B.scardovii* JCM12489	3.16	2418	64.6	56	9	AP012331.1

The average size of a bifidobacterial genome is 2.2 Mb, although there is considerable size variation, for example *B. indicum* LMG11587 harbors a genome with a size of 1.73 Mb, wheras *B. scardovii* JCM12489 possesses a genome of 3.16 Mb in length. Bifidobacterial genomes typically encode 52–58 tRNA genes per genome, although there are exceptions, e.g., the genome of *B. longum* subsp. *infantis* ATCC15697 encompasses 79 tRNA-encoding genes. The number of rRNA operons within bifidobacterial genomes typically ranges from two to five, and it has been suggested that the number of rRNA operons present on a genome is correlated to the adaptation of a particular species to environmental conditions (Klappenbach et al., 2000). The G+C content of complete bifidobacterial genomes ranges from 59.2% (*B. adolescentis*) to 64.6% (*B. scardovii*), while the average gene number contained by a bifidobacterial genome is 1825 (Table 1). The three species *B. indicum, B. coryneforme,* and *B. animalis* possess the lowest number of genes, consistent with their small genome size (Lee et al., 2008; Ventura et al., 2014).

## Impact on health and disease

A diverse microbial community has evolved to adapt and survive in the human GIT and is commonly referred to as the gut microbiota (Guarner and Malagelada, 2003). The large intestine can contain up to 10^12^ bacterial cells/g of luminal content making this the most densely populated area of the gastrointestinal tract (Simon and Gorbach, 1984). Members of the gut microbiota interact with their (human) host in a variety of ways, thereby making them innocuous commensals, opportunistic pathogens or health-promoting or probiotic micro organisms (Guarner and Malagelada, 2003). Probiotics are defined as “live microorganisms that, when administered in adequate amounts, confer a health benefit on the host” (FAO/WHO, 2001; Hill et al., 2014), and research into the activities of purported health-promoting bacteria has increased substantially over the last 20 years (Leahy et al., 2005). Probiotic agents have been investigated in many clinical and animal model-based studies; however, we will summarize just a limited number of studies that specifically relate to bifidobacteria. Bifidobacteria have been commercially exploited as probiotic agents due to their associated health benefits and GRAS (Generally Recognised As Safe) status (Picard et al., 2005).

### Bifidobacteria and colorectal cancer

Several studies have investigated the potential of bifidobacteria to prevent and/or treat colorectal cancer. The majority of studies base their findings on murine models, and results suggest that a combination of prebiotics and bifidobacteria may reduce the occurrence of carcinogen-induced cancerous cells in mice (Sekine et al., 1985; Rowland et al., 1998; Rafter et al., 2007; Le Leu et al., 2010). For example, it was shown that *B. animalis* displays anti-mutagenic activity during growth in MRS broth thereby antagonizing the action of the carcinogen 2-amino-3-methylimidazo [4, 5-f] quinolone (Tavan et al., 2002). It has also been demonstrated under *in vivo* and *in vitro* conditions that a *B. longum* and a *B. breve* strain provide protection of DNA from induced damage by carcinogens, and inhibit the genotoxic effect of two different carcinogens when tested in a rat model (Pool-Zobel et al., 1996).

### Bifidobacteria and diarrhoea

The use of bifidobacteria to treat various gastrointestinal disorders has also been reported. For example, successful treatment of diarrhea following administration of *B. longum* subsp. *infantis* CECT 7210 and *B. breve* K-110 was found to be due to inhibition of rotavirus, the predominant cause of sporadic diarrhea in infants (Bae et al., 2002; Chenoll et al., 2015). Another example involves a double-blind study investigating whether oral treatment with a commercial probiotic formula containing *B. bifidum* and *Streptococcus thermophiles* would reduce antibiotic-associated diarrhea in infants. This study found that there was a significant reduction in incidences of diarrhea for those infants fed the probiotic supplemented formula supplemented (Corrêa et al., 2005).

### Bifidobacteria and necrotizing entercolitis

A recent study reported lower incidences of necrotizing enterocolitis in preterm neonates following routine administration of *B. breve* M-16V (Patole et al., 2016). Administration of *B. breve* M-16V in association with breast-feeding was shown to be associated with a lower incidence of necrotising enterocolitis in neonates born before 34 weeks gestation, and, although not statistically significant, a lower incidence in this disease was reported for neonates born at a gestation age of less than 28 weeks (Patole et al., 2016).

### Bifidobacteria and inflammatory bowel disease

Although, the exact mechanism of action is not understood, reduction in the symptoms of inflammatory bowel disease following treatment by probiotic strains has been reported (Venturi et al., 1999). Patients suffering from ulcerative colitis were given a probiotic preparation that includes three *Bifidobacterium* strains, four *Lactobacillus* strains and one *S. thermophilus* strain. Fifteen out of the 20 patients remained in remission throughout the trial, suggesting that administration of this bacterial cocktail is beneficial in maintaining remission from ulcerative colitis (Venturi et al., 1999; Gionchetti et al., 2000).

### Bifidobacteria and colon regularity

A number of studies have reported improvements in colon regularity following ingestion of fermented milk products that contain *B. animalis* (Marteau et al., 2002; Guyonnet et al., 2007; Meance et al., 2011). Two studies have associated the administration of certain bifidobacterial strains with the alleviation of constipation (Kumemura et al., 1992; Kleessen et al., 1997). However, further investigation is needed in order to identify the precise mechanism(s) of action elicited by bifidobacteria in the prevention and treatment of constipation (Leahy et al., 2005).

### Bifidobacteria and competitive exclusion

Bifidobacteria have also been reported to prevent gastrointestinal infections by competitive exclusion of pathogens based on common binding sites on epithelial cells (Duffy et al., 1994a,b; Perdigon et al., 1995; Picard et al., 2005; Gueimonde et al., 2007). Administration of high levels of bifidobacteria was shown to decrease the viable counts of *Clostridium perfringens*, a known producer of undesirable toxins (Tanaka et al., 1983).

## Bifidobacteria and functional foods

The inclusion of micro-organisms in the human diet has been on-going for thousands of years (Leahy et al., 2005). Throughout history the most common form of administration of microorganisms was through fermented dairy products and this is still the case today (Leahy et al., 2005). Certain lactic acid bacteria, in particular certain members of the genus *Lactobacillus*, and members of the *Bifidobacterium* genus make up the vast majority of the functional ingredients present in currently commercialized probiotic food products (Salminen and Wright, 1998; Ouwehand et al., 2002). Prebiotics have been defined as “selectively fermented ingredients that allow for specific changes, both in the composition and/or activity of the gastrointestinal microflora that confer benefits upon host well-being and health” (Hijova et al., 2009). This definition has been revisited several times since it was first introduced in 1995, although these alternative definitions are in agreement that prebiotics need to be “specific” or “selective” (Gibson and Roberfroid, 1995; Roberfroid et al., 2010; Rastall and Gibson, 2015). In a recent review the definition of prebiotics was revisited and proposed as follows: “a prebiotic is a non-digestible compound that, through its metabolisation by microorganisms in the gut, modulates composition and/or activity of the gut microbiota, thus conferring a beneficial physiological effect on the host” (Bindels et al., 2015).

The newly proposed definition moves away from the requirement of “specific effect” and puts forward the arguments that: (i) our knowledge does not allow for a reliable differentiation between beneficial and detrimental members of the microbiota, (ii) a diverse community is essential for intestinal homeostasis and host physiology, (iii) the metabolic benefits assigned to prebiotics do not require a “selective” fermentation, and (iv) community-wide molecular approaches have revealed that established prebiotics are not as specific as previously assumed (Bindels et al., 2015).

One outcome from the fermentation of prebiotics by the gut microbiota is the production of short chain fatty acids (SCFAs), such as acetate, butyrate and propionate (Broekaert et al., 2011). SCFA production in the GIT results in a lower pH, improved availability of calcium and magnesium, and inhibition of potentially pathogenic bacteria (Teitelbaum and Walker, 2002; Wong et al., 2006). Both bifidobacteria and lactobacilli produce acetate (and lactate), thus contributing to the SCFA-mediated health effects of prebiotics, although these two microorganisms do not produce butyrate and/or propionate (Fukuda et al., 2011; Bindels et al., 2015). The latter SCFAs are produced by members of the *Bacteroides* phylum and the *Clostridium* clusters XIVa and IV (Louis et al., 2010; Reichardt et al., 2014; Bindels et al., 2015). Furthermore, a recent study has demonstrated that acetate produced by *B. longum* NCC2705 acts as an essential co-substrate for butyrate production and growth by *Eubacterium rectale* ATCC 33656 (Rivière et al., 2015).

Non-digestible oligosaccharides (NDOs), typically obtained from complex carbohydrates or enzymatically produced from disaccharides, represent a group of glycans that include various prebiotics (Grootaert et al., 2007). Examples of this are fructo-oligosaccharides (FOS) and galacto-oligosaccharides (GOS), which are among the best documented and most commonly used prebiotics on the European and Japanese markets (Grootaert et al., 2007). The prebiotic effects of FOS, GOS, inulin and lactulose have been thoroughly assessed in human trials and many studies suggest that these carbohydrates are selective by increasing bifidobacterial numbers and decreasing the numbers of *E. coli* and enterococci (Menne et al., 2000; Kolida et al., 2002; Bosscher et al., 2006; Kapiki et al., 2007; Davis et al., 2010; Veereman-Wauters et al., 2011; Walton et al., 2012).

Due to the professed prebiotic effects of arabinoxylan (AX) and its derivatives arabinoxylo-oligosaccharides (AXOS) and xylo-oligosaccharides (XOS), these carbohydrates have in recent times enjoyed increasing scientific interest (Broekaert et al., 2011). The bifidogenic effect of AX has been confirmed in a number of *in vitro* studies (Van Laere et al., 2000; Crittenden et al., 2002), while the ability of bifidobacteria to metabolize XOS and AXOS in pure culture has also been demonstrated (Jaskari et al., 1998; Van Laere et al., 2000; Crittenden et al., 2002; Palframan et al., 2003; Moura et al., 2007). AXOS consumption amongst members representing eleven different bifidobacterial species suggests that AXOS metabolism is strain dependent and rather complex (Riviere et al., 2014). In this study, five different AXOS utilization clusters were identified based on principal component analysis of the different arabinose substituent and/or xylose backbone consumption patterns. The first and largest cluster (Cluster I) was composed of 15 different strains representing seven different species (*B. adolescentis, B. angulatum, B. bifidum, B. breve, B. dentium, B. longum,* and *B. thermophilum*). Strains within this cluster were unable to utilize the substitutions or xylan backbone of AXOS, although some strains were able to utilize the monosaccharides xylose and arabinose. Cluster II was composed of eight *B. longum* strains that were unable to utilize the xylan backbone, yet were able to utilize the arabinose substitutions on AXOS (both mono- and di-substituted), as well as the arabinose and xylose monosaccharides. Members of the third cluster (Cluster III), encompassing 10 strains representing six different species (*B. adolescentis, B. angulatum, B. longum, B. animalis, B. gallicum,* and *B. pseudolongum*), were shown to metabolize the xylan backbone of AXOS, albeit only up to xylotetraose, while eliciting no or limited utilization of the AXOS substitutions. Cluster IV contains two *B. longum* strains that share the ability to completely utilize AXOS, whereas the only member of Cluster V, *B. catenulatum* LMG 11043, was shown to display non-preferential degradation of XOS and a broad degradation pattern of arabinose substitutions (Riviere et al., 2014). A study investigating the *in vitro* fermentation of wheat-derived AX (AX-W) by human fecal microbiota reported that fermentation of AX-W was associated with the proliferation of bifidobacteria, lactobacilli and eubacteria (Hughes et al., 2007).

Several *in vivo* studies have also confirmed the bifidogenic effect of AX. An *in vivo* study in humanized rats demonstrated that long chain AX specifically stimulates the abundance of several different bacterial species in the cecum (relative bifidobacterial abundance in the cecum of the control group was 0.03 ± 0.01%, compared to 2.81 ± 1.46% in the group that were fed long chain AX; Van Den Abbeele et al., 2011). The findings of this latter study were validated by a recent study which detected the presence of two different *B. longum* species during the fermentation of long chain AX in an *in vitro* model of the proximal colon (Truchado et al., 2015). Another *in vivo* study found that when (high-fat) diet-induced obese mice were fed an AX-supplemented diet, a significant increase in caecal bifidobacterial numbers was observed (Neyrinck et al., 2011). Along with this increase in caecal bifidobacteria, AX supplementation restored (some of) the high-fat diet-induced changes to the microbial community.

Synbiotics are mixtures of one or more probiotics combined with one or more prebiotics (Patel and Dupont, 2015). Numerous *in vivo* studies have been conducted aimed at investigating the efficacy of bifidobacteria-based synbiotics in the treatment of gastrointestinal diseases and conditions. One such study investigated the synbiotic effect of *B. animalis* subsp. *lactis* B94 in combination with inulin on acute infectious diarrhea in children. Patients were administered the synbiotic agent once a day for five days and stool was examined for infectious agents such as rotavirus, *Salmonella, Shigella, Campylobacter, Cryptosporidium, Adenovirus, Entamoeba histolytica,* and *Clostridium difficile*. A marked decrease in the number of diarrhea stools was reported after 3 days of administration for the synbiotic group as compared to the control group, particularly for patients with rotavirus infection (Islek et al., 2014). A clinical trial investigated the effects of consumption of a synbiotic on the symptoms of Crohn's disease (Steed et al., 2010). The synbiotic, comprised of *B. longum,* inulin, and oligofructose, was consumed by patients twice daily over a 6 month period, and significant improvements in clinical outcomes were reported including a reduction in some activity indices of Crohn's disease (Steed et al., 2010). As a third example, the beneficial effect of a *B. breve* strain plus GOS synbiotic was investigated with regards to ulcerative colitis. The bifidobacterial strain was ingested three times a day whereas GOS was consumed once a day for 1 year. The clinical status of the treatment group significantly improved such as a marked improvement in colonoscopy scores and significant decreases in inflammatory markers. Furthermore, although no significant change in bifidobacterial numbers for those consuming the symbiotic was noticed, reduced fecal counts of *Bacteriodaceae* and reduced fecal pH was noted (Ishikawa et al., 2011).

## Bifidobacterial carbohydrate metabolism

The human genome is predicted to encode just eight glycosyl hydrolases (GHs) that are directly linked to carbohydrate digestion. Therefore, many complex dietary carbohydrates remain un-digested and end up in the colon where they may be degraded by members of the microbiota (El Kaoutari et al., 2013).

The human GIT is home to complex microbial community that encompasses approximately 100-fold more genes than the number of genes present in the host genome (Backhed et al., 2005). Colonization of the human GIT, which is believed to occur immediately after birth, is influenced by various factors such as the method of delivery (i.e., vaginal or cesarean), type of feeding (breast-fed or formula-fed), exposure to antibiotics, frequency, and nature of diseases and hygiene conditions (Fanaro et al., 2003). Bifidobacteria dominate the total gut bacterial population in healthy breast-fed infants (Harmsen et al., 2000; Favier et al., 2002; Leahy et al., 2005), although this dominance decreases following weaning (Ventura et al., 2004). During adult life the bifidobacterial population stabilizes to represent 3–6% of the total gut microbial population, whereas in elderly (>65 years) the bifidobacterial numbers usually decline with age (Hopkins et al., 2001; Satokari et al., 2003).

The abundance and make-up of the gut microbiota is (among others) dependent on the diet of its host, and members of the microbiota have evolved effective mechanisms to utilize available nutrients (Vaughan et al., 2005; Ju-Hoon and O'Sullivan, 2010). Digestible and simple sugars such as lactose and sucrose are metabolized in the upper gut by the host and bacteria such as lactobacilli, a prevalent inhabitant of the upper GIT (Ganong, 2005; Vaughan et al., 2005). A diverse set of non-digestible carbohydrates are metabolized in the lower gut, including complex plant-derived polysaccharides (e.g., pectin, gums, hemicellulose, and xylans), host-derived carbohydrates (such as mucin and glycosphingolipids), and extracellular polysaccharides that are produced by members of the gut microbiota (Hooper et al., 2002; Korakli et al., 2002; Pokusaeva et al., 2011a). It is therefore not surprising that on average more than 12% of the annotated open reading frames within bifidobacterial genomes is predicted to encode enzymes involved in carbohydrate metabolism (Milani et al., 2014). In fact a recent study performed on the genome sequences from the type strains of each of the 47 *Bifidobacterium* (sub)species found that 5.5% of the core bifidobacterial genomic coding sequences (BifCOGs) is associated with carbohydrate metabolism (Milani et al., 2015).

Bifidobacteria present in the infant gut are presumed to metabolize human milk oligosaccharides (HMOs), and the genomes of *B. bifidum* and *B. longum* subsp. *infantis* are indeed tailored toward HMO metabolism (Sela et al., 2008; Duranti et al., 2015). However, other bifidobacterial species such as *B. breve* and *B. longum* subsp. *longum* are also commonly present in the infant gut. Although, they do not encode the same HMO catabolic arsenal found in *B. bifidum* and *B. longum* subsp. *infantis,* they can degrade certain HMOs and may also scavenge on carbohydrates that are released by other (bifido)bacteria (Egan et al., 2014a; Chaplin et al., 2015). After weaning the composition of the bifidobacterial population changes toward species capable of adapting to the metabolism of plant-derived sugars. For example, *B. longum* subsp. *longum* and *B. adolescentis* can utilize such diet-derived carbohydrates, while *B. bifidum* may shift its HMO-metabolic abilities toward mucin degradation (Schell et al., 2002; Turroni et al., 2010; Sela, 2011; Duranti et al., 2014; Egan et al., 2014a).

**Figure 1:**
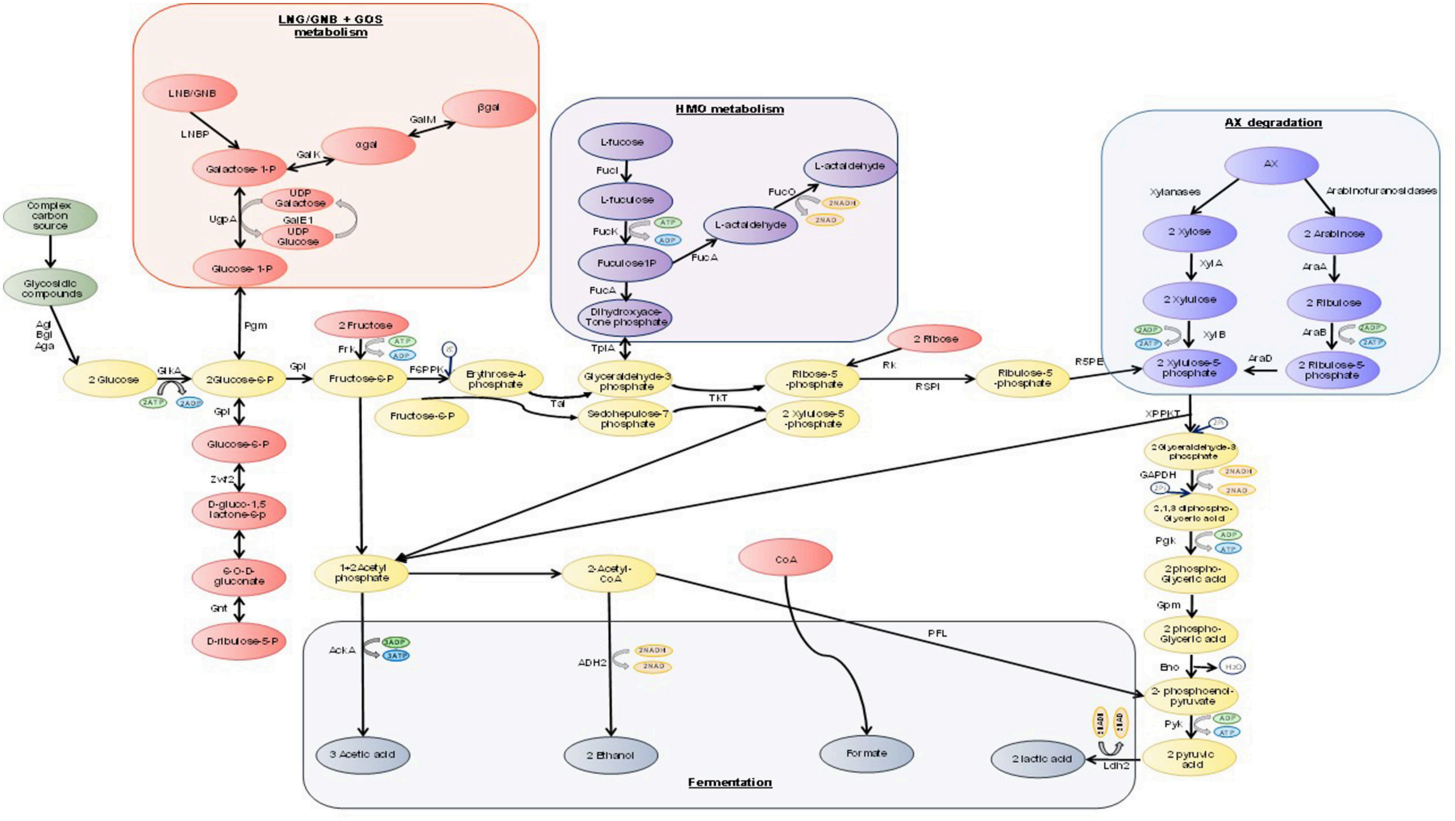
**A schematic representation of carbohydrate degradation through the “bifid shunt” in bifidobacteria**. Abbreviations: AckA, acetate kinase; Adh2, Aldehyde-alcohol dehydrogenase 2; Aga, α-galactosidase; Agl, α-glucosidase; AraA, L-arabinose isomerase; AraB, Ribulokinase; AraD, L-ribulose-5-phosphate 4-epimerase; Bgl, β-glucosidsae; Eno, enolase; GalE1, UDP-glucose 4-epimerase; GalK, galactokinase; GalM, glactose mutarotase; GAPDH, glyceraldehyde-3-phosphate dehydrogenase C; GlkA, glucokinase; Gnt, 6-phosphogluconate dehydrogenase; Gpi, glucose 6-phosphate isomerase; Gpm, phosphoglycerate mutase; FrK, frucktokinase; F6PPK, fructose-6-phodphoketolase; FucI, L-fucose isomerase; FucK, L-fuculose kinase; FucA, L-fuculose-1-phosphate aldose; FucO, lactaldehyde reductase; Ldh2, lactate dehydrogenase; LNBP, lacto-N-biose phosphorylase; Pgk, phosphoglyceric kinase; Pgm, phosphoglucomutase; Pfl, formate acetyltransferase; Pyk, pyruvate kinase; Rk, ribokinase; R5PI, ribose-5-phosphate isomerase; R5PE, ribulose-5-phosphate epimerase; Tal, transaldase; Tkt, transketolase; TpiA, trisephosphate isomerase; UgpA, UTP-glucose-1-phosphate uridylyltransferase; XPPKT, xylulose-5-phosphate/fructose-6-phosphate phosphoketolase; XylA, xylose isomerase; XylB, xylulose kinase; Zwf2, glucose-6-phosphate 1-dehydrogenase; Pi, phosphate (based on a figure from a previous review Pokusaeva et al., 2011a).

Prediction of the number of complete pathways used by bifidobacteria to degrade simple and complex sugars has been performed. The species *B. biavatti* specifies the largest number of pathways (14 complete pathways), whereas members of the species *B. bombi, B. crudilactis, B. longum* subsp. *infantis, B. minimum,* and *B. ruminantium* specifying just nine complete pathways (Milani et al., 2015). Bifidobacteria lack a number of key enzymes involved in the Emden-Meyerhof Parnas (EMP) pathway, instead, bifidobacteria metabolize hexose sugars through a metabolic pathway named the “bifid shunt” which is centered around the key enzyme, fructose-6-phosphoketolase (EC 4.1.2.2) (Figure 1; De Vries and Stouthamer, 1967; De Vuyst et al., 2014). Furthermore, the action of additional enzymes allows for a variety of carbon sources (including pentose sugars) to be channeled through this pathway (Pokusaeva et al., 2011a). Fermentation through the bifid shunt is quite advantageous for bifidobacteria as this pathway allows for the production of more energy from carbohydrates compared to that produced by the EMP fermentative pathway (Salminen and Wright, 1998; Palframan et al., 2003). The bifid shunt theoretically yields 2.5 ATP moles from every 1 mole of glucose fermented, as well as 1.5 mole of acetate and 1 mole of lactate (Palframan et al., 2003). The ratios of acetate to lactate can be influenced, however, by the particular carbohydrate being fermented as well as the growth phase and bifidobacterial species being examined (Palframan et al., 2003). Furthermore, rapid consumption of an energy source was shown to result in the production of large amounts of lactate and low amounts of acetate, ethanol and formate, whereas less lactate is produced along with an increase in production of acetate, formate and ethanol when the energy source is consumed at a slow(er) rate (Figure 1; Van Der Meulen et al., 2004, 2006a,b; Falony et al., 2009).

### Carbohydrate uptake strategies by bifidobacteria

Bifidobacteria internalize carbohydrates by ATP-dependent ABC transporters, proton symporters or phosphoenolpyruvate-phosphotransferase systems (PEP-PTS) (Turroni et al., 2012). ABC transporters couple ATP hydrolysis to efficient internalization of sugars and appear to represent the primary carbohydrate transport systems for bifidobacteria (Ventura et al., 2007; Davidson et al., 2008; Jojima et al., 2010). PEP-PTS systems allow the concomittant transport and phosphorylation of carbohydrates, while they may also be involved in the regulation of various metabolic pathways (Postma et al., 1993). The PTS component of the system is involved in the internalization and concomitant phosphorylation of carbohydrates, while PEP acts as the (indirect) phosphate donor to the recipient carbohydrate (Ventura et al., 2007). These systems are found in many bacteria and have also been identified in most, but not all, bifidobacterial genomes (Postma et al., 1993; Pokusaeva et al., 2011a). The action of the PEP-PTS system was demonstrated experimentally in bifidobacteria whereby a PEP-PTS system from *B. breve* UCC2003 was found to be involved in the internalization of glucose (Degnan and Macfarlane, 1993). Since then a number of PEP-PTS systems have been identified and investigated in other bifidobacterial species (Lorca et al., 2007; Parche et al., 2007; Barrangou et al., 2009; Turroni et al., 2012; O'Connell Motherway et al., 2013).

The *B. longum* subsp. *longum* DJO10A and NCC2705 genomes are predicted to represent 10 and 13 different ABC transporters, respectively, responsible for the uptake of carbohydrates, while they each encode just a single glucose-specific PEP-PTS system (Lorca et al., 2007; Parche et al., 2007). Analysis of the *B. bifidum* PRL2010 genome sequence revealed that this strain encodes two ABC transporters, four PEP-PTS systems and four secondary transporters that are expected to transport mono- and disaccharides (Turroni et al., 2012). Transcriptional analysis revealed that one ABC transporter and two of the PEP-PTS systems are associated with the internalization of degradation products of host-derived glycans, in particular those that are found in mucin. The ABC transporters identified in *B. bifidum* PRL2010 were found to be linked to the uptake of monosaccharides such as glucose, ribose, fructose and galactose or disaccharides such as turanose (Turroni et al., 2012). Like *B. bifidum* PRL2010, *B. breve* UCC2003 is also predicted to encode four PEP-PTS systems and it has been shown experimentally that one system in *B. breve* UCC2003 is a fructose-inducible fructose/glucose uptake system (O'Connell Motherway et al., 2013). However, *B. breve* UCC2003 typically employs ABC-type transporters for the uptake of carbohydrates (Pokusaeva et al., 2011a, O'Connell Motherway et al., 2013; Egan et al., 2014b). In contrast, the strain *B. animalis* subsp. *lactis* B1-04 does not possess any PEP-PTS system and only encodes two copies of an ATP-binding protein linked to carbohydrate internalization (Barrangou et al., 2009). This low number of carbohydrate uptake systems may be a reflection of genome decay due to the commercial exploitation of *B. animalis* subsp. *lactis*, for which purpose it is extensively cultivated in nutritionally rich media (Lee et al., 2008).

### Bifidobacterial glycosyl hydrolases

Carbohydrates can be modified by a range of different enzymes including hexosyl- and phosphotransferases, hydrolases and isomerizes (Pokusaeva et al., 2011a). In the presence of water, glycosyl hydrolases (GHs) hydrolyse the glycosidic bond between two or more sugars or alternatively between a carbohydrate and a non-carbohydrate moiety (Pokusaeva et al., 2011a). GHs are assigned the enzyme commission or EC number EC 3.2.1x whereby the first three numbers indicate that these particular enzymes cleave glycosyl linkages, with the assignment of the final number being based on the target substrate or mechanism of action displayed by the enzyme. Members of one GH family may not only exhibit different substrate specificity, but may also exhibit a different mode of action (Van Den Broek et al., 2008). Classification of GHs can be found at http://www.cazy.org/Glycoside-Hydrolases.html (Lombard et al., 2014). For bifidobacteria GHs are the most prevalent group of carbohydrate-modifying enzymes and since the publication of the first bifidobacterial genome more information on these GHs has become available (Van Den Broek et al., 2008).

None of the carbohydrate active GHs encoded by the human genome appear to be involved in the breakdown of FOS, GOS, XOS, inulin, or arabinoxylan (Guarner and Malagelada, 2003; El Kaoutari et al., 2013). A recent study investigated the distribution of different carbohydrate-active enzymes among 177 bacterial genomes of the human microbiome, including genomes of 12 members of the *Actinobacteria* phylum, half of which were bifidobacteria (El Kaoutari et al., 2013). Polysaccharide lyases and GHs accounted for 59% of all carbohydrate-active enzymes identified. From these observations it can be said that the microbiota endows metabolic activities that make up for the paucity of GHs encoded by the human genome (El Kaoutari et al., 2013).

According to the current GH classifications, *B. scardovii* and *B. indicum* LMG11587 are predicted to encode the highest (126 GHs) and lowest number of GHs (25 GHs), respectively, among the currently sequenced bifidobacterial genomes (Table 2). Classification according to the Carbohydrate Active Enzymes (CAZy) system revealed that 3385 genes belonging to the bifidobacterial pan-genome are predicted to represent carbohydrate active enzymes, including members of 57 GH families, 13 GT families and 7 carbohydrate esterases (Milani et al., 2015). Those enzymes belonging family GH13 are the most commonly found in bifidobacterial genomes and are known to be active against a wide range of carbohydrates including the plant-derived carbohydrates starch and related substrates, trehalose, stachyose, raffinose, and melibiose (Pokusaeva et al., 2011a; Milani et al., 2015). The bifidobacterial glycobiome contains a large number of enzymes belonging to the families GH29, GH95, GH20, GH112, GH38, GH125, GH101, and GH129 which are involved in the degradation of host-derived glycans. Members of the *B. scardovii, B. longum* subsp. *infantis,* and *B. bifidum* species in particular encode the most extensive set of enzymes active against host-derived glycans (Milani et al., 2015). Unlike other species, those bifidobacterial species isolated from honey/bumblebees encode a very limited set of GH13 representatives. However, these species specify a larger set of GH43 and GH3 enzymes. These families are active against plant-derived polysaccharides, such are arabino(xylan), (arabino)galactan, and cellodextran (Milani et al., 2015). Furthermore, a substantial number of genes encoding putative arabino(xylan)-degrading enzymes are present in certain bifidobacterial genomes, for example *B. longum* subsp. *longum* NCC2705, hinting at the importance of these enzymes toward the colonization of these microorganisms in the GIT (Schell et al., 2002; Van Den Broek et al., 2008).

Table 2**Bifidobacterial glycosyl hydrolases**.

**Table:** 

**CAZy family (GH)**	**Substrate specificities**	*****B. adolescentis*** 15703**	*****B. adolescentis*** 22L**	*****B. adolescentis*** BBMN23**	*****B. angulatum*** DSM20098**	*****B. animalis*** A6**	*****B. animalis*** RH**	*****B. animalis*** subsp. ***animalis*** ATCC25527**	*****B. animalis*** subsp. ***lactis*** ADO11**	*****B. animalis*** subsp. ***lactis*** ATCC27673**	*****B. animalis1*** subsp. ***lactis*** B420**	*****B. animalis*** subsp. ***lactis*** Bb12**	*****B. animalis1*** subsp. ***lactis*** BF052**	*****B. animalis*** subsp. ***lactis*** Bi-07**	*****B. animalis*** subsp. ***lactis*** BI-04**	*****B. animalis*** subsp. ***lactis*** BI12**	*****B. animalis*** subsp. ***lactis*** BLC1**	*****B. animalis*** subsp. ***lactis*** CNCM I-2494**	*****B. animalis*** subsp. ***lactis*** DSM 10140**	*****B. animalis*** subsp. ***lactis*** KLDS2.0603**	*****B. animalis*** subsp. ***lactis*** V9**
1	β-glucosidase, β-galactosidase	2	2	0	1	1	1	1	1	1	1	1	1	1	1	1	1	1	1	1	1
2	β-galactosidase	3	4	2	2	2	2	2	2	2	2	2	2	2	2	2	2	2	2	2	2
3	β-glucosidase, β-hexosaminidase, β-D-glucosideglucohydrolase,	6	5	6	3	3	3	3	3	2	3	3	3	3	3	3	3	3	3	3	3
5	β-mannosidase, β-glucosidase, β-exoglucanase	2	2	2	0	1	1	1	1	2	1	1	1	1	1	1	1	1	1	1	1
8	Xylanase	1	1	1	1	0	0	0	0	0	0	0	0	0	0	0	0	0	0	0	0
13	α-1, 4-glucosidase, amylopullulanase, sucrose Phosphorylase, α-amylase	13	16	17	13	13	13	13	11	10	13	14	13	13	11	10	11	13	11	13	11
23	Transglycosylase	3	2	2	2	2	1	2	2	2	2	2	2	2	2	2	2	2	2	2	2
25	β-N-acetylmuramidase	1	2	3	1	2	2	1	2	1	2	2	2	2	2	2	2	2	2	2	2
26	Endo-1, 4-β-mannosidase	1	1	0	0	0	0	0	0	0	0	0	0	0	0	0	0	0	0	0	0
27	α-galactosidase	1	0	0	0	0	0	0	0	0	0	0	0	0	0	0	0	0	0	0	0
30	β-D-xylosidase, endo-1, 6-β-glucosidase, Glucosylceramidase	1	1	1	0	2	2	1	2	1	2	2	2	2	2	2	2	2	2	2	2
31	α-xylosidase	1	1	2	2	0	0	0	0	0	0	0	0	0	0	0	0	0	0	0	0
32	β-fructofuranosidase, sucrose-6-phosphate hydrolase	2	2	2	2	1	1	1	1	1	1	1	1	1	1	1	1	1	1	1	1
35	β-galactosidase	1	0	0	0	0	0	0	0	0	0	0	0	0	0	0	0	0	0	0	0
36	α-galactosidase, raffinose synthase,	2	3	2	2	3	3	3	3	3	3	3	3	3	3	3	3	3	3	3	3
38	α-mannosidase	1	1	1	0	0	0	0	0	0	0	0	0	0	0	0	0	0	0	0	0
39	β-xylosidase	0	1	0	0	0	0	0	0	0	0	0	0	0	0	0	0	0	0	0	0
42	β-galactosidase	4	3	3	2	2	2	0	2	1	2	2	2	2	2	2	2	2	2	2	2
43	Endo-1, 5-α-L-arabinosidase, α-L-arabinofuranosidase, Endo-1,4-β-xylanase, β-1, 4-xylosidase	7	7	8	5	3	3	3	3	3	3	3	3	3	3	3	3	3	3	3	3
51	α-L-arabinofuranosidase	2	1	1	1	1	1	1	1	1	1	2	1	1	1	1	1	1	1	1	1
77	4-α-glucanotransferase	2	2	2	2	2	2	2	2	2	2	2	2	2	2	2	2	2	2	2	2
94	Cellobiose-phosphorylase	0	1	1	0	1	1	0	1	1	1	1	1	1	1	0	1	1	1	1	1
120	β-xylosidase	1	1	1	1	0	0	0	0	0	0	0	0	0	0	0	0	0	0	0	0
121	β-galactosidase	0	0	1	0	0	0	0	0	0	0	0	0	0	0	0	0	0	0	0	0
127	β-L-arabinofuranosidase	1	2	2	0	1	1	0	1	1	1	1	1	1	1	1	1	1	1	1	1
Total		58	61	60	40	40	39	34	38	35	40	42	40	40	38	36	38	40	38	40	38

**Table:** 

**CAZy family (GH)**	**Substrate specificities**	***B. asteroides* PRL2011**	***B. bifidum* ATCC29521**	***B. bifidum* BGN4**	***B. bifidum* PRL2010**	***B. bifidum* S17**	***B. breve* 12L**	***B. breve* 6589b**	***B. breve* ACS-071-V-Sch8b**	***B. breve* DSM20213**	***B. breve* JCM7017**	***B. breve* JCM7019**	***B. breve* NCFB2258**	***B. breve* S27**	***B. breve* UCC2003**	***B. catenulatum* DSM 16992**	***B. coryneforme* LMG 18911**	***B. dentium* Bd1**	***B. dentium* JCM1195**	***B. indicum* LMG 11587**
1	β-glucosidase, β-galactosidase	0	2	1	1	1	1	1	2	1	2	3	1	2	2	0	1	2	2	1
2	β-galactosidase	2	3	3	3	3	2	3	4	2	2	4	3	3	5	4	0	6	6	0
3	β-glucosidase, β-hexosaminidase, β-D-glucosideglucohydrolase,	4	1	1	1	1	3	6	5	6	4	4	5	4	3	7	4	11	12	4
4	6-phospho-β-glucosidase	1	0	0	0	0	0	0	0	0	0	0	0	0	0	0	0	0	0	0
5	β-mannosidase, β-glucosidase, β-exoglucanase	1	0	0	0	0	2	2	2	2	2	2	2	2	2	2	0	4	4	0
13	α-1,4-glucosidase, amylopullulanase, sucrose phosphorylase, α-amylase	4	7	7	9	7	14	14	13	12	13	12	13	12	14	9	2	14	14	2
16	Adenine glycosylase	0	1	0	0	0	0	0	0	0	0	0	0	0	0	0	0	0	0	0
18	Chitinase	0	0	0	0	0	1	2	1	0	1	1	1	2	1	0	0	0	0	0
20	β-hexosaminidase	0	4	4	4	4	1	1	1	1	1	1	1	1	1	0	0	0	0	0
23	Transglycosylase	2	2	3	2	2	4	3	2	3	3	2	3	3	2	3	2	3	3	2
25	β-N-acetylmuramidase	0	3	1	2	1	1	1	1	1	2	1	1	1	1	1	1	2	2	1
26	Endo-1, 4-β-mannosidase	0	0	0	0	0	0	0	0	0	0	0	0	0	0	0	0	2	2	0
27	α-galactosidase	1	0	0	0	0	0	0	0	0	0	0	0	0	0	0	0	1	1	0
28	Galacturan 1, 4-α-galacturonidase, pectinesterase	2	0	0	0	0	0	0	0	0	0	0	0	0	0	0	0	0	0	0
29	α-L-fucosidase	1	1	1	1	1	0	0	0	0	0	0	0	0	0	0	0	1	1	0
30	β-D-xylosidase, endo-1, 6-β-glucosidase, Glucosylceramidase	3	0	0	0	0	0	1	0	0	0	1	0	0	1	1	3	1	1	3
31	α-xylosidase	3	0	0	0	0	0	0	0	0	1	1	1	1	2	1	1	4	4	1
32	β-fructofuranosidase, sucrose-6-phosphate hydrolase	1	2	0	0	0	1	1	1	1	2	1	1	2	2	2	2	2	2	2
33	Sialidase	0	4	3	3	3	1	1	1	1	1	1	1	1	1	0	0	0	0	0
35	β-galactosidase	0	0	0	0	0	0	0	0	0	0	1	0	0	1	0	0	0	0	0
36	α-galactosidase, raffinose synthase,	2	1	1	1	1	3	2	2	2	2	3	2	2	2	4	2	5	5	2
38	α-mannosidase	2	0	0	0	0	2	2	2	2	2	3	3	2	3	1	0	0	0	0
39	β-xylosidase	0	0	0	0	0	0	0	0	0	0	0	0	0	0	1	0	0	0	0
42	β-galactosidase	2	2	2	2	1	2	2	3	2	3	2	2	2	2	3	2	4	4	2
43	Endo-1, 5-α-L-arabinosidase, α-L-arabinofuranosidase, Endo-1,4-β-xylanase, β-1, 4-xylosidase	9	3	2	2	2	0	1	1	1	1	0	0	0	0	7	3	12	12	3
51	α-L-arabinofuranosidase	2	1	1	1	1	0	0	0	0	0	0	0	0	0	3	1	2	2	0
53	Endogalactanase	0	0	0	0	0	1	1	1	0	1	0	1	1	1	0	0	2	2	0
59	β-galactosidase/galactocerebrosidase	0	0	0	0	0	0	1	0	0	0	0	0	0	0	0	0	0	0	0
65	Kojibiose phosphorylase	1	0	0	0	0	0	0	1	0	0	0	1	1	1	0	1	0	0	1
67	α-glucuronidase	1	0	0	0	0	0	0	0	0	0	0	0	0	0	0	0	0	0	0
77	4-α-glucanotransferase	0	1	1	1	1	2	2	2	2	2	2	2	2	2	2	0	2	2	0
78	α-L-rhamnosidase	1	0	0	0	0	0	1	0	0	0	1	0	0	0	0	1	1	1	1
84	α-L-rhamnosidase	0	2	2	2	2	0	0	0	0	0	0	0	0	0	0	0	0	0	0
85	Endo-β-N-acetylglucosaminidase D	0	0	0	0	0	0	1	0	0	1	1	1	0	1	0	0	0	0	0
89	α-N-acetylglucosaminidase, β-N-hexosaminidase	0	1	1	1	1	0	0	0	0	0	0	0	0	0	0	0	0	0	0
94	Cellobiose-phosphorylase	0	0	0	0	0	0	0	0	0	0	0	0	0	0	0	0	1	1	0
95	α-L-fucosidase	0	1	1	1	1	1	1	1	1	1	1	1	1	1	0	0	0	0	0
101	endo-α-N-acetylgalactosaminidase	0	1	1	1	1	0	0	0	0	0	0	0	0	0	0	0	0	0	0
105	rhamnogalacturonyl hydrolase, d-4,5-unsaturated β-glucuronyl hydrolase	1	0	0	0	0	0	0	0	0	0	0	0	0	0	0	0	0	0	0
110	Exo-α-galactosidase	0	1	1	1	1	0	0	0	0	0	0	0	0	0	0	0	0	0	0
112	Lacto-N-biose phosphorylase	0	2	2	2	2	1	1	1	1	1	1	1	1	1	0	0	0	0	0
115	xylan α-1, 2-glucuronidase, α-(4-O-methyl)-glucuronidase	2	0	0	0	0	0	0	0	0	0	0	0	0	0	0	0	0	0	0
120	β-xylosidase	0	0	0	0	0	0	0	0	0	0	0	0	0	0	1	0	0	0	0
121	β-galactosidase	0	0	0	0	0	0	0	0	0	0	0	0	0	0	1	0	1	1	0
123	Glycosphingolipid β-N-acetylgalactosaminidase	0	1	1	1	1	0	0	0	0	0	0	0	0	0	0	0	0	0	0
125	Exo-α-1, 6-mannosidase	0	0	0	0	0	1	1	1	1	1	1	1	1	1	0	0	1	1	0
127	β-L-arabinofuranosidase	0	0	0	0	0	1	1	0	1	0	2	1	1	0	3	0	2	2	0
129	α-N-acetylgalactosaminidase	0	1	1	1	1	1	1	0	1	1	1	1	1	1	0	0	0	0	0
NC	Unknown	0	0	0	0	0	1	1	1	1	1	1	1	1	1	0	0	1	1	0
Total		48	48	41	43	39	47	55	49	45	51	54	51	50	55	57	26	87	88	25

**Table:** 

**CAZy family (GH)**	**Substrate specificities**	***B. kashiwanohense* JCM 15439**	***B. kashiwanohense* PV20-2**	***B. longum* 105-A**	***B. longum* BXY01**	***B.* lognum DJO10A**	***B. longum* NCC2705**	***B. longum* subsp. *infantis* 157F**	***B. longum* subsp. *infantis* ATCC15697**	***B. longum* subsp. *infantis* JCM1222**	***B. longum* subsp. *longum* BBMN68**	***B. longum* subsp. *longum* F8**	***B. longum* subsp. *longum* GT15**	***B. longum* subsp. *longum* JCM1217**	***B. longum* subsp. *longum* JDM301**	***B. longum* subsp. *longum* KACC91563**	***B. pseudocatenulatum* D2CA**	***B. pseudocatenulatum* DSM20438**	***B. pseudolongum* PV8-2**	***B. scardovii* JCM12489**	***B. thermophilum* RBL67**
1	β-glucosidase, β-galactosidase	2	1	1	0	0	0	0	1	1	0	0	0	0	0	0	1	0	1	1	0
2	β-galactosidase	4	2	2	3	2	2	2	2	3	1	2	2	2	2	2	2	4	3	7	0
3	β-glucosidase, β-hexosaminidase, β-D-glucosideglucohydrolase,	5	6	6	3	3	3	5	2	2	4	3	5	4	3	4	5	5	2	10	1
5	β-mannosidase, β-glucosidase, β-exoglucanase	1	2	2	2	2	2	2	2	2	2	2	2	1	2	1	2	2	0	2	3
8	Xylanase	2	0	0	1	0	0	0	0	0	0	0	0	0	1	0	1	2	0	0	0
10	Endoxylanase	1	0	0	0	0	0	0	0	0	0	0	0	0	0	0	0	0	0	0	0
13	α-1, 4-glucosidase, amylopullulanase, sucrose phosphorylase, α-amylase	9	8	8	12	13	8	11	8	9	13	10	12	13	12	13	15	17	13	12	14
16	Adenine glycosylase	0	0	0	0	0	0	0	0	0	0	0	0	0	0	0	0	0	0	1	0
18	Chitinase	1	1	1	0	0	0	1	1	1	0	0	0	0	0	0	0	1	0	1	1
20	β-hexosaminidase	1	1	1	1	1	1	1	3	3	1	1	1	1	1	1	1	1	0	3	0
23	Transglycosylase	4	1	1	2	4	3	3	2	2	2	3	3	2	2	3	1	3	2	3	2
25	β-N-acetylmuramidase	1	1	1	0	1	0	1	4	4	1	1	0	1	0	0	1	1	1	4	1
26	Endo-1, 4-β-mannosidase	0	0	0	0	0	0	0	0	0	0	0	0	0	0	0	0	1	0	2	0
27	α-galactosidase	1	0	0	1	1	1	1	0	0	1	0	1	1	1	1	1	1	0	1	0
28	Galacturan 1,4-α-galacturonidase, pectinesterase	0	0	0	0	0	0	0	0	0	0	0	0	0	0	0	0	0	0	2	0
29	α-L-fucosidase	2	0	0	1	0	0	0	3	3	0	0	0	0	1	0	0	0	0	2	0
30	β-D-xylosidase, endo-1, 6-β-glucosidase, Glucosylceramidase	1	0	0	1	1	1	1	0	0	2	0	1	2	1	1	2	1	1	2	0
31	α-xylosidase	3	2	2	1	3	3	2	0	0	2	3	1	2	1	2	3	2	1	6	1
32	β-fructofuranosidase, sucrose-6-phosphate hydrolase	2	2	2	2	1	1	1	2	2	1	1	2	1	2	1	1	1	1	2	2
33	Sialidase	0	0	0	0	0	0	0	2	2	0	0	0	0	0	0	0	0	0	1	0
35	β-galactosidase	1	0	0	1	0	0	0	0	0	0	0	0	0	1	0	0	0	0	1	0
36	α-galactosidase, raffinose synthase,	2	2	2	2	2	1	1	1	1	2	2	1	2	2	2	3	3	4	12	2
38	α-mannosidase	1	1	1	3	3	3	2	2	2	2	2	2	0	3	0	0	0	0	2	0
39	β-xylosidase	0	0	0	0	0	0	0	0	0	0	0	0	0	0	0	0	0	0	3	0
42	β-galactosidase	3	3	3	4	3	2	3	3	3	2	2	3	2	4	2	3	4	2	6	1
43	Endo-1, 5-α-L-arabinosidase, α-L-arabinofuranosidase, Endo-1,4-β-xylanase, β-1, 4-xylosidase	9	6	6	10	11	9	7	1	1	11	7	7	11	10	8	12	15	7	17	0
50	β-agarase	0	0	0	1	0	0	0	0	0	0	0	0	0	1	0	0	0	0	0	0
51	α-L-arabinofuranosidase	2	1	1	4	5	5	4	1	1	3	4	4	4	4	4	2	3	1	6	1
53	Endogalactanase	0	0	0	1	1	1	1	0	1	1	1	1	1	1	1	0	0	0	0	0
59	β-galactosidase/galactocerebrosidase	0	0	0	0	0	0	0	0	0	0	0	0	0	0	0	1	0	0	1	0
65	Kojibiose phosphorylase	0	0	0	0	0	0	1	0	0	0	0	0	0	0	0	0	0	0	0	0
76	α-1, 6-mannanase	0	0	0	0	0	0	0	0	0	0	0	0	0	0	0	0	0	0	1	0
77	4-α-glucanotransferase	2	2	2	2	2	2	2	2	2	2	2	2	2	2	2	2	2	2	2	2
78	α-L-rhamnosidase	0	1	1	0	0	0	0	0	0	0	0	0	0	0	0	0	0	0	2	0
85	Endo-β-N-acetylglucosaminidase D	0	0	0	1	1	1	1	0	0	1	1	1	0	1	0	0	0	0	0	0
94	Cellobiose-phosphorylase	0	0	0	0	0	0	0	0	0	0	0	0	0	0	0	0	0	1	1	0
95	α-L-fucosidase	1	1	1	1	0	0	0	1	1	0	0	0	0	1	0	0	1	0	0	0
101	endo-α-N-acetylgalactosaminidase	0	0	0	0	1	1	1	0	0	1	0	1	1	0	1	0	0	0	0	0
112	Lacto-N-biose phosphorylase	0	0	0	1	1	1	1	1	1	1	1	1	1	1	1	0	0	0	1	0
115	xylan α-1, 2-glucuronidase, α-(4-O-methyl)-glucuronidase	0	0	0	0	0	0	0	0	0	0	0	0	0	0	0	0	0	0	1	0
120	β-xylosidase	1	0	0	1	2	0	1	0	0	0	1	1	0	1	1	1	2	0	1	0
121	β-galactosidase	0	0	0	0	0	1	1	0	0	1	1	1	1	0	1	0	1	0	0	0
125	Exo-α-1, 6-mannosidase	0	0	0	1	1	1	1	0	1	1	1	1	0	1	0	0	0	0	1	0
127	β-L-arabinofuranosidase	1	0	0	1	1	2	2	0	0	2	2	2	2	1	2	0	1	0	2	0
129	α-N-acetylgalactosaminidase	0	0	0	1	1	1	1	1	1	0	1	1	1	1	1	0	0	0	1	0
130	β-mannose phosphorylase	0	0	0	0	0	0	0	0	0	0	0	0	0	0	0	0	0	0	1	0
NC	Unknown	0	0	0	0	0	0	0	1	0	0	0	0	1	0	0	0	0	0	2	0
Total		63	44	44	65	67	56	61	46	49	60	54	59	59	64	55	60	74	42	126	31

Enzymes active against such arabinose- and xylose-containing carbohydrates have been characterized from *B. longum, B. adolescentis, B. animalis* subsp. *lactis,* and *B. breve*, and were first described in *B. adolescentis* (Van Laere et al., 1997; Lagaert et al., 2011). Seven bifidobacterial arabinofuranosidases have currently been characterized belonging to the families GH43 and GH51, five of which are produced by *B. adolescentis*, while two originate from *B. longum* (Van Laere et al., 1999; Margolles and De Los Reyes-Gavilan, 2003; Lagaert et al., 2010; Lee et al., 2011; Suzuki et al., 2013). Five intracellular xylanases have been characterized in bifidobacteria to date for both *B. breve* and *B. adolescentis* (Lagaert et al., 2007, 2011; Hyun et al., 2012; Amaretti et al., 2013). It is worth noting that all of the 14 currently characterized bifidobacterial arabino(xylan)-degrading enzymes are (predicted to be) intracellular. Due to their sizes, arabino(xylan)s cannot be transported inside the cell and the lack of extracellular enzymes specified by these bifidobacterial strains hints that they may rely on the extracellular hydrolytic activity of other members of the gut microbiota. Furthermore, the degradation of arabino(xylan) by extracellular enzymes has been observed previously for some bifidobacterial strains, and such extracellular enzymes may therefore be of significant interest as they are expected to provide an ecological advantage in the GIT (Riviere et al., 2014).

Investigations have been performed in order to determine those bifidobacterial GHs that are expected to be either secreted into the environment or associated with the cell surface. The majority of GHs located within the bifidobacterial pan-genome are predicted to be intracellular with 10.9% of GHs predicted to be secreted. The members of the family GH13 represent the largest proportion of such (predicted) secreted GHs, followed by members of the GH43 and GH51 families (Milani et al., 2015).

## Carbohydrate cross-feeding by bifidobacteria

Several recent studies have investigated the impact on the gut microbiome by bifidobacterial cross-feeding of carbohydrates. Various studies have demonstrated that some members of the bifidobacterial community can co-operate in order to degrade large and complex polysaccharides into more simple sugars which are in turn then available to other members of the gut microbiota (De Vuyst and Leroy, 2011). This has been demonstrated for plant derived polysaccharides and also for host-derived carbohydrates such as mucin (Milani et al., 2014, 2015; Egan et al., 2014a,b; Turroni et al., 2016).

Bifidobacteria were found to shape the microbiome of the murine gut either through direct action or by cross-feeding activities (Turroni et al., 2016). This study demonstrated that the addition of two or more bifidobacterial strains resulted in the enhanced levels of persistence of such strains within the murine cecum. Furthermore, members of this genus were capable of modulating gene expression toward an increase in glycan metabolism encompassing both host glycans and diet derived carbohydrates. The bifidobacterial strains had a further influence over the production of SCFAs (Turroni et al., 2016).

## Control of bifidobacterial carbohydrate metabolism

Carbon catabolite repression (CCR) is a regulatory system present in many bacteria in which the expression or activity of proteins involved in the utilization or uptake of available carbon sources is inhibited by the presence of a preferred carbon source (Postma et al., 1993; Saier and Ramseier, 1996). There is evidence that a CCR mechanism is operating in bifidobacteria, although as of yet a CCR-type regulatory system has not been described for any member of this genus. The first report on CCR-related metabolism in bifidobacteria was in *B. animalis* subsp. *lactis* (Trindade et al., 2003). This study reported an induction in sucrose metabolizing activities when this strain was grown on sucrose, raffinose or oligofructose, whereas a repression in the same metabolic activities was reported for growth on glucose (Trindade et al., 2003). An apparent reverse CCR was reported for *B. longum* NCC2705 in that glucose transport was repressed when lactose was present in the growth medium (Parche et al., 2006). There are two separate reports of CCR-like regulation in *B. breve* UCC2003 (Ryan et al., 2005; Pokusaeva et al., 2010). The first study reported that transcription of the *rbs* operon, responsible for ribose metabolism, is induced when *B. breve* UCC2003 is grown on ribose, whereas transcription of this operon is not induced (or repressed) when the strain is grown on a combination of ribose and glucose (Pokusaeva et al., 2010). The second study on *B. breve* UCC2003 demonstrated that when grown on sucrose or Actilight (a commercial source of short chain FOS), transcription of the *fos* operon, which is involved in the metabolism of FOS, is induced (Ryan et al., 2005). However, this operon was not induced (or repressed) when *B. breve* UCC2003 is grown on a combination of sucrose and glucose, or sucrose and fructose (Ryan et al., 2005).

Transcriptional repressors, such as LacI-type transcriptional regulators, are DNA binding proteins that physically interact with a specific DNA sequence, called the operator, in the vicinity of a regulated promoter, thereby preventing the binding of an RNA polymerase and the initiation of transcription (Ravcheev et al., 2014). Transcriptional repressors may also cause a “road block” for the DNA polymerase thus preventing the progression of transcription (Ravcheev et al., 2014). A substantial number of these repressor proteins have been identified in bifidobacterial genomes, e.g., *B. longum* NCC2705 is predicted to encode 22 LacI-type transcriptional repressors. The presence of a sugar-binding motif in each of these predicted 22 LacI-type proteins indicates that they are predicted to be involved in the regulation of carbohydrate metabolism (Schell et al., 2002). Six different LacI-type regulators have been characterized in *B. breve* UCC2003 and include LacI_fos_, GalR, CldR, and RbsR, which regulate transcription of the *fos* operon, the galactan utilization cluster, the cellodextrin utilization cluster, and the ribose metabolism cluster, respectively (Ryan et al., 2005; Pokusaeva et al., 2010, 2011b; O'Connell Motherway et al., 2011a). More recently, two *B. breve* UCC2003-encoded LacI-type transcriptional repressors, named MelR1 and MelR2, were shown to control the melezitose utilization cluster (O'Connell et al., 2014).

Other carbohydrate-dependent regulatory systems exist, for example, the RafR regulator is a transcriptional activator of the raffinose metabolism gene cluster in *B. breve* UCC2003 (O'Connell et al., 2014), while in the same bacterium a GntR-type transcriptional repressor was shown to control transcription of the large *nag/nan* gene cluster, thereby regulating metabolism of sialic acid (Egan et al., 2015).

## Biotechnology of bifidobacteria

Research into bifidobacterial metabolism has led to the prebiotic concept which in turn confers health benefits to the human host by stimulating the metabolism and activity of bifidobacteria in the GIT (Hijova et al., 2009). However, the actual mechanisms of action that are responsible for such probiotic effects are far from fully understood (Sun et al., 2012). The gold standard approach to investigate the role of a single gene and its products is by site-directed mutagenesis and subsequent phenotypic analysis of the generated mutant(s) (Sun et al., 2012). Unfortunately, bifidobacteria are rather recalcitrant to artificial DNA acquisition methods which means that genetic modification of bifidobacteria has up until recently proven to be impossible. This part of the review will focus on genetic approaches and techniques aimed at improving the genetic accessibility of bifidobacteria.

## Bifidobacterial mutagenesis strategies

There are a small number of reports on targeted gene inactivation in bifidobacteria, although in recent years a number of techniques have been developed resulting the successful inactivation of genes in bifidobacteria. In this part of the review, we describe the fundamental concept of each strategy and discuss some of benefits and pitfalls associated with each.

### Single-crossover plasmid insertion

**Figure 2:**
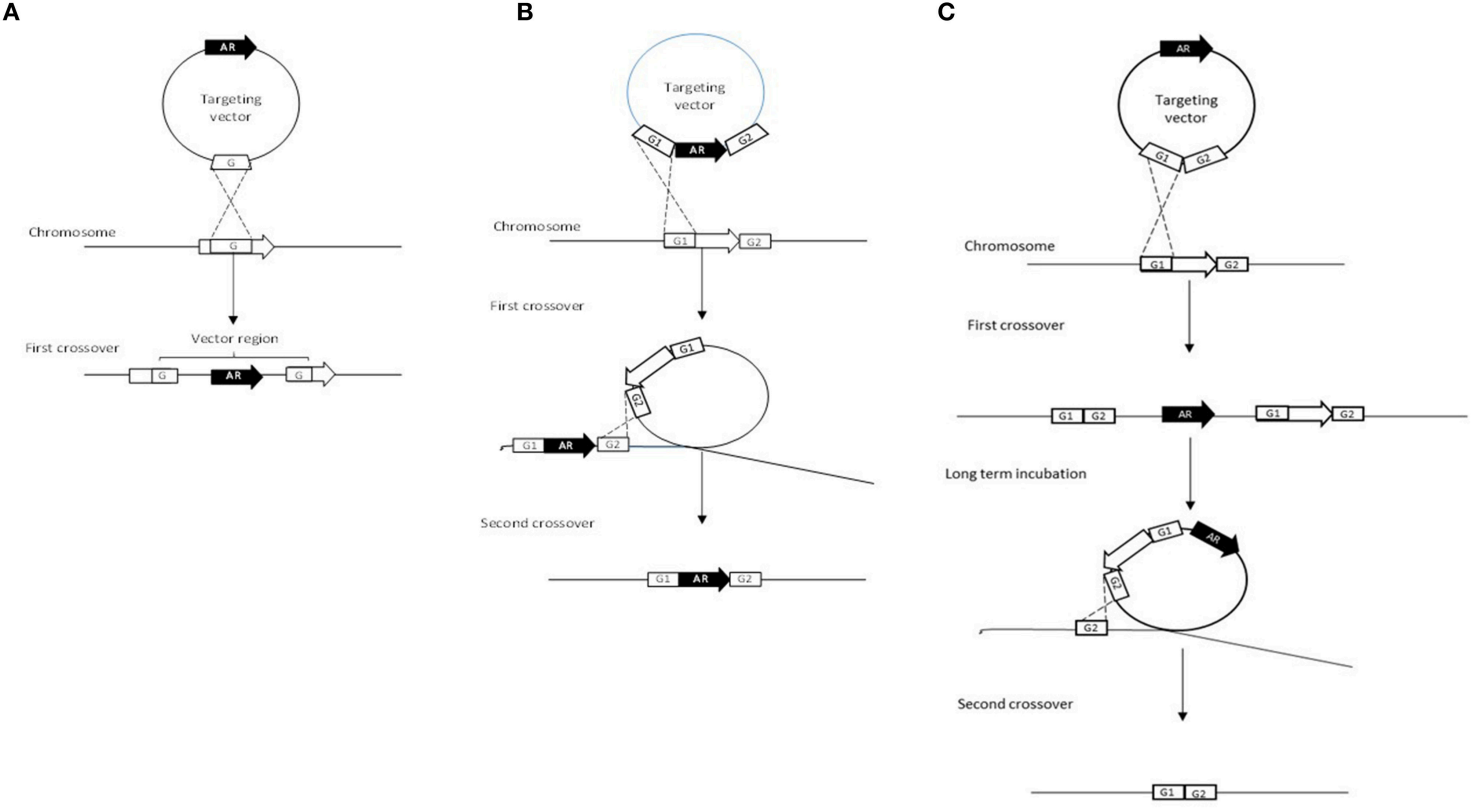
**Schematic representation of targeted gene inactivation approaches**. The targeted gene is indicated by an open arrow and homologous regions are indicated as open squares and given the names G1 and G2. The antibiotic resistance gene is indicated as a black arrow with the letters AR. **(A)** represents the single-crossover plasmid insertion, **(B)** illustrates the double-crossover gene disruption and finally **(C)** illustrates the double-crossover markerless gene deletion approach. (Figure was adapted from Fukiya et al., 2012).

This method involves the use of a non-replicating plasmid to select for homologous recombination events (Figure 2A). The first successful mutation created in a bifidobacterial strain was in the gene *apuB* using this single-crossover plasmid insertion approach whereby a combination of plasmids facilitated the conditional replication of the non-replicative plasmid pORI19 (Table 3; O'Connell Motherway et al., 2008). Although, it successfully led to the inactivation of the *apuB* gene, this method was quite time consuming and laborious (Fukiya et al., 2012).

**Table 3:** **Summary of bifidobacterial mutagenesis strategies**.

**Concept**	**Benefits**	**Pitfalls**	**Example**
**SINGLE-CROSSOVER PLASMID INSERTION**			
• Non-replicative plasmid insertion	• Reproducible	• Prior knowledge of the strain is essential	• Disruption of *galA* and *apuB* genes in *B. breve* UCC2003 (O'Connell Motherway et al., 2009)
• Single cross-over homologous recombination	• Requires only a single transformation round	• Successful knock-outs left with antibiotic marker	
• Internal region of target gene and marker		• Requires high transformation efficiencies	
		• Unstable mutations	
**DOUBLE-CROSSOVER GENE DELETION**			
• Non-replicative plasmid insertion	• Stable mutation	• Successful knock-outs left with antibiotic marker	• Disruption of *BL0033* in *B. longum* NCC2705 (Fukuda et al., 2011)
• Double crossover homologous recombination	• Gene target is deleted	• Time-consuming and laborious- multiple transformations and extensive screening of transformants	
• 5′ and 3′ regions of target gene separated by marker		• Requires high transformation efficiencies	
**DOUBLE CROSS-OVER MARKER-LESS GENE DELETION**			
• Non-replicative plasmid insertion	• Stable mutation	• Time-consuming and laborious- multiple transformations and extensive screening of transformants	• Disruption of *aga* in *B. longum* 105-A (Hirayama et al., 2012)
• Double cross-over homologous recombination	• Gene deletion		
• 5′ and 3′ regions of target gene adjacent to marker	• Successful knock-outs left without antibiotic marker		
• Marker-less gene disruption			
**TEMPERATURE SENSITIVE (TS) PLASMID FOR GENE DISRUPTION**			
• Ts plasmid unable to replicate at high temperatures	• Does not require high transformation efficiencies	• Time-consuming and laborious- multiple transformations and extensive screening of transformants	• Disruption of *apuB* in *B. breve* UCC2003 (O'Connell Motherway et al., 2008)
• Ts plasmid contains homologous sequence and marker			
• Homologous recombination			
**TRANSPOSON MUTAGENESIS FOR GENE DISRUPTION**			
• Random mutagenesis with Transposome complex	• Generation of a large mutant bank	• Successful knock-outs left with antibiotic marker	• Creation of a mutant library in *B. breve* UCC2003 (Ruiz et al., 2013)
• Large number of gene disruptions	• High throughput screening of bank	• Requires high transformation efficiencies	
• Antibiotic marker			

As already discussed above, by-pass of native R-M systems and the single-crossover plasmid insertion approach using a non-replicating plasmid has been successfully and repeatedly employed for mutagenesis (O'Connell Motherway et al., 2009, 2011b, 2013; Fouhy et al., 2013; Christiaen et al., 2014; Egan et al., 2014a,b, 2015; O'Connell et al., 2014).

### Double-crossover and double-crossover markerless gene deletion

Double-crossover gene disruption was first demonstrated in *B. longum* NCC2705 (Table 3; Fukuda et al., 2011). This method makes use of a non-replicating plasmid and a double-cross over event in order to create a gene deletion (Fukiya et al., 2012). The non-replicative vector harbors two homologous regions of the target gene between which an antibiotic resistance gene is inserted (Figure 2B). During the first crossover event, homologous recombination occurs between one of the two homologous regions on the vector and on the chromosome. The second (desired) crossover occurs between the second homologous region on the vector and the chromosome, and results in the replacement of the wild-type allele with the mutated one and an antibiotic marker (Figure 2B). In the case of *B. longum* NCC2705, this approach allows for the successful deletion of (part of) the gene *BL0033,* a solute binding protein of an ABC transporter that is highly induced when *B. longum* NCC2705 is grown on fructose (Fukuda et al., 2011).

The double-crossover marker-less strategy has been patented and draws some similarities to the double-crossover gene disruption approach (Table 3, Figure 2C; Arigoni and Delley, 2013). Like the previous approach, a non-replicative plasmid is used for the first crossover, however, in this case the antibiotic marker is located beside the mutated targeted gene (Figure 2C). The second crossover event occurs during long-term sub-culturing of the first crossover integrants. As illustrated in Figures 1, 2C and as described previously for the double-crossover gene disruption, a markerless gene deletion can be generated following a second crossover. An amended version of this method was used to successfully create a marker-less gene deletion in *B. longum* 105-A which involved the conditional replication of a vector plasmid (Table 3; Hirayama et al., 2012).

The plasmid used for the homologous recombination events is a conditionally replicating plasmid due to a deletion of the gene encoding the replication protein RepA. This plasmid can only replicate in bifidobacteria when the RepA protein is provided *in trans.* The regions flanking the target gene to be deleted are cloned into the conditional replication vector (Hirayama et al., 2012). Once the plasmid has integrated into the target gene via the first homologous recombination or cross-over event, a second plasmid that encodes RepA is introduced which induces the conditional replication of the integrated plasmid (Figure 2C). During the second cross-over and excision, the integrated plasmid is expected to be excised along with the target gene. Second cross-over recombinants that lost the first plasmid can be selected for using appropriate antibiotic markers as the second plasmid is incompatible with the conditional replicating vector (Hirayama et al., 2012).

The double cross-over approaches are more time consuming due to multiple transformations and extensive screening for positive mutants, although the improved double crossover gene deletion system aims to address this (Table 3; Fukiya et al., 2012). For single cross-over mutants it is theoretically possible for additional cross-over events to occur between the homologous regions left in the target gene thus resulting in the excision of the integrated plasmid (Fukiya et al., 2012). It is also worth noting that the marker-less method is a superior approach as it does not leave an antibiotic resistance gene, the presence of which may cause polar effects affecting genes down-stream of the mutated gene (Table 3; Fukiya et al., 2012).

### Homologous recombination mediated by a temperature sensitive plasmid

The temperature sensitive (Ts) plasmid strategy has been applied successfully to various microorganisms in order to create gene knock-outs (Hamilton et al., 1989; Maguin et al., 1992; Biswas et al., 1993; Takamatsu et al., 2001; Fuchs et al., 2006; Chen et al., 2011). The advantage of this approach over some of the other strategies described above, is that Ts plasmids do not require high transformation efficiencies. Therefore, this would be an ideal and widely applicable approach for (transformation recalcitrant) bifidobacterial strains (Table 3; Sakaguchi et al., 2012).

**Figure 3:**
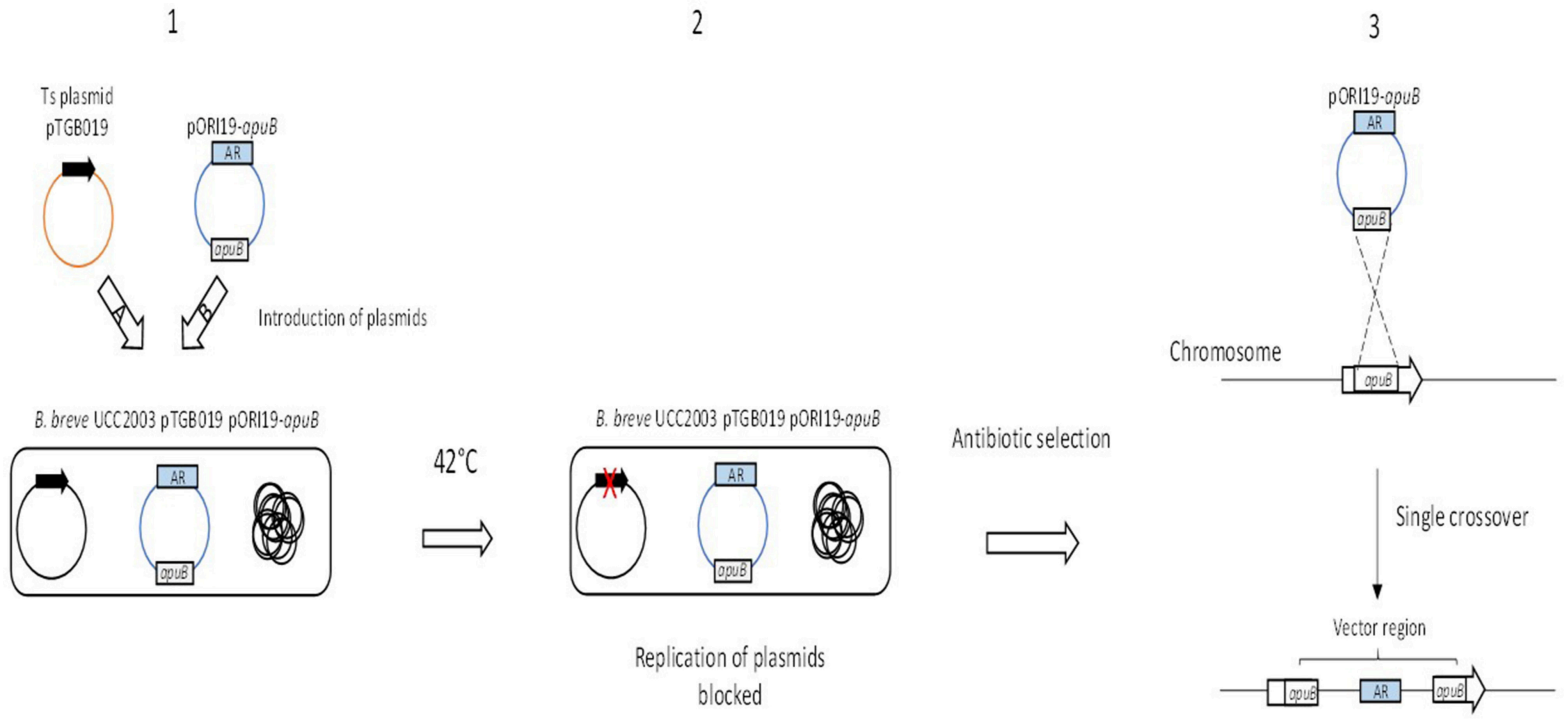
**Schematic representation of insertion mutagenesis using a temperature sensitive plasmid**. The targeted gene is indicated by the text *apuB* in the open gray box and the antibiotic resistance gene is indicated as a blue box with the letters AR. The black arrow represents the replication gene *repA* and the red-cross indicates a gene that is non-functional. Step **1** illustrates the introduction of (a) the plasmid pTGB019 into *B. breve* UCC2003 followed by (b) the introduction of the non-replicative plasmids pORI19-apuB. Step **2** illustrates a shift in temperature resulting in the blocked replication of pTGB019 and pORI19-apuB as a consequence of a non-functional *repA* gene. Finally in Step **3**, presence of antibiotic selects for integration of pORI19-derivatives into the desired site on the *B. breve* UCC2003 genome.

The successful creation of insertion mutants using a Ts plasmid has been achieved in *B. breve* UCC2003, *B. longum* 105-A and *B. longum* NCC2705 (O'Connell Motherway et al., 2008; Sakaguchi et al., 2012). A plasmid harboring *repA* was first introduced into *B. breve* UCC2003 followed by the introduction of a non-replicating plasmid that harbored an internal fragment of the target gene, *apuB* (Table 3, Figure 3; O'Connell Motherway et al., 2008). Once both plasmids were successfully introduced, growth of *B. breve* UCC2003 was shifted to 42°C, thus preventing further replication of the *repA*-containing plasmid. As a consequence this also blocks the replication of the pORI19-derivative. Selection on antibiotic results in the integration of the pORI19-derivative into the *B. breve* UCC2003 genome at the site of the *apuB* gene (Figure 3; O'Connell Motherway et al., 2008). This temperature-sensitive plasmid approach was also used to create insertion mutants in *B. longum* 105-A and *B. longum* NCC2705 (Sakaguchi et al., 2012). This method was applied to create an insertion mutant in the strain *B. longum* 105-A using *pyrR* as the gene target and was further validated by re-creating a gene deletion in the gene *BL0033* in the strain *B. longum* NCC2705 (Table 3; Sakaguchi et al., 2012).

### Transposons for mutagenesis in bifidobacteria

A recent study has described the creation of a mutant library in *B. breve* UCC2003 using a Tn5-based transposon mutagenesis system (Ruiz et al., 2013). This study is the first to demonstrate the application of random-mutagenesis technology in bifidobacteria (Table 3). This approach involved the construction of a tetracycline-resistant Tn5 transposon and preparation of a transposome complex that was subsequently introduced into *B. breve* UCC2003 cells by electroporation (Ruiz et al., 2013). Screening of the mutant bank led to the identification of mutants that display defective growth on selected carbohydrates, such as *Bbr_0010* which encodes a β-galactosidase involved in the metabolism of lactose (Ruiz et al., 2013).

The benefit of this method is that it can be used for high-throughput screening in order to identify genes that are fundamental for a given phenotype (Judson and Mekalanos, 2000). However, similarly to targeted insertion mutant systems, high transformation efficiencies are crucial for this system to be effective (Ruiz et al., 2013).

## Conjugation in bifidobacteria

Conjugation can also be used by bacteria to transfer genetic material, as was first described in *E. coli* and involving cell-to-cell contact (Lederberg and Tatum, 1946). Conjugation-based techniques have been widely described for Gram-negative bacteria, though less so for Gram-positive bacteria (Schroder and Lanka, 2005).

Currently, transformation by electroporation is the most popular method used to genetically manipulate bifidobacteria. Shortfalls maybe overcome by the use of a conjugation-based approach as it holds a number of advantages over transformation (Dominguez and O'Sullivan, 2013). Firstly, unlike electroporation, the size of the vector does not affect conjugation efficacy (Szostková and Horáková, 1998; Isaacs et al., 2011). Secondly, the presence of R-M systems is not an issue for conjugative methods as during conjugation DNA is transferred as a single strand from donor to recipient, in which form it is insensitive to most R-M systems (Dominguez and O'Sullivan, 2013).

The first, though unsuccessful, attempt at conjugative transfer in bifidobacteria was reported in 1998 (Shkoporov et al., 2008). A recent publication describes a conjugative gene transfer method and the first successful transfer of DNA between *E. coli* and different *Bifidobacterium* species (Dominguez and O'Sullivan, 2013). Based on the RP4 conjugative machinery of *E. coli* WM3064 (pBB109), the *E. coli-Bifidobacterium* shuttle vector pDOJHR-WD2 was constructed. This plasmid was transformed into *E. coli* WM3064 harboring pBB109. This latter plasmid encodes the relaxase function to catalyze nicking at the *oriT* site on pDOJHR-WD2, while RP4, which is integrated into the *E. coli* WM3064 chromosome, specifies Tra functions (Dominguez and O'Sullivan, 2013). Successful transfer of pDOJHR-WD2 from *E. coli* to members of four different bifidobacterial species was reported with an efficiency ranging from 1.8 × 10^−4^ to 7.5 × 10^−6^ transconjugants per recipient (Dominguez and O'Sullivan, 2013).

The recent sequencing of the *B. breve* JCM 7017 genome led to the discovery of a 190 kb megaplasmid designated as pMP7017 (Bottacini et al., 2015). Analysis of the plasmid sequence led to the identification of genes that are predicted to encode conjugative machinery. Sequence analysis of pMP7017 led to the observation that, although the overall G+C content is akin to that of its host, it appears that this megaplasmid was formed from the co-integration of two separate modules (Bottacini et al., 2015). The functionality of the conjugative mechanism was demonstrated by the successful transfer of pMP7017 from *B. breve* JCM7017 to *B. breve* and *B. longum* representatives (Bottacini et al., 2015).

The recent development of conjugation-based gene transfer systems and the discovery of the first native conjugative bifidobacterial plasmid is quite significant (Dominguez and O'Sullivan, 2013; Bottacini et al., 2015), and may assist in future tool development for the genetic modification of bifidobacteria.

## Microbe-host interactions

Insertion mutagenesis has proven to be a fundamental tool in the development of functional genomics in bifidobacteria. Two studies in particular demonstrate how the use of insertion mutagenesis can reveal how candidates genes are involved in the beneficial effects conferred to the host by bifidobacteria (Fukiya et al., 2012).

In the first example, Fukuda et al. combined comparative genomics, gene expression profiling and gene knock-outs to demonstrate that acetate production by the strain *B. longum* subsp. *longum* JCM 1217 is linked to the protection of host epithelial cells from lethal doses of the shiga toxin, Stx2 (Fukuda et al., 2011). Early observations highlighted that germ-free mice pre-colonized with *B. longum* subsp. *longum* JCM 1217 and subsequently infected with enterohaemorrhagic *E. coli* O157:H7 (EHEC O157:H7), survived better than germ-free mice that were infected with EHEC O157:H7. In contrast, the same survival was not observed when, prior to infection with EHEC O157:H7, mice were colonized with *B. adolescentis* JCM 1275. Comparative genomic analysis performed between protective *B. longum* subsp. *longum* and non-protective *B. adolescentis* implicated an ABC-type transporter system being involved in protection, after which a knock-out mutation was created in the gene encoding the corresponding solute binding protein using a double crossover gene disruption approach (Fukuda et al., 2011). Due to the consequently lower levels of acetate produced, the mutant strain was unable to protect mice from EHEC O157:H7 infection, whereas when the non-protective *B. adolescentis* strain expressed the ABC transporter cluster this resulted in moderate increases in mouse survival following EHEC O157:H7 infection as well as increased acetate production.

The second example of a functional microbe-host interaction study is the identification of candidate genes directly responsible for the colonization of *B. breve* UCC2003 in a murine colonization model (O'Connell Motherway et al., 2011b). Genome and *in vivo* transcriptome analyses of *B. breve* UCC2003 revealed that a gene cluster, responsible for the production of so-called type IVb or tight adherence (Tad) pili,is required for host colonization (O'Connell Motherway et al., 2011b).

As well as investigating candidate genes directly involved in host-microbe interactions, insertion mutagenesis has also been exploited in functional genomic studies investigating the physiological characteristics of bifidobacteria, such as carbohydrate metabolism (O'Connell Motherway et al., 2011a; O'Connell et al., 2013, 2014; Egan et al., 2014b, 2015).

## Conclusion

It is well established that bifidobacteria confer positive health benefits to their host via their metabolic activities. The availability of complete bifidobacterial genomes and corresponding comparative analysis allows for the identification of mechanisms underlying bifidobacterial metabolic activity. Carbohydrate utilization studies and identification of metabolic pathways also provides fundamental information allowing for the identification of novel and effective prebiotic compounds.

Plant-derived and host derived carbohydrates have been shown to stimulate the growth of some bifidobacterial species. To identify and obtain full knowledge of the genes implicated in carbohydrate degradation and utilization, characterization and mutagenesis of candidate genes is required. However, bifidobacteria are notoriously recalcitrant to genetic modification.

It is therefore essential that future studies continue to address the shortage of effective molecular tools. The development of these tools is essential to unravel the underlying molecular mechanisms that explain how bifidobacteria interact with their human host.

## Author contributions

All authors listed, have made substantial, direct and intellectual contribution to the work, and approved it for publication.

## Funding

This publication has emanated from research conducted with the financial support of Science Foundation Ireland (SFI) under Grant Number SFI/12/RC/2273. AO was supported by an enterprise partnership scheme of the Irish Research Council.

### Conflict of interest statement

The authors declare that the research was conducted in the absence of any commercial or financial relationships that could be construed as a potential conflict of interest.
